# Social Media Addiction and Its Association With Psychological Distress and Academic Performance Among Libyan Medical Students: A Cross-Sectional Study

**DOI:** 10.7759/cureus.101152

**Published:** 2026-01-09

**Authors:** Aseel A Beetru, Rehab A Sherlala, Asad M Aljared, Bodor A Jarjar, Hoda M Tawel

**Affiliations:** 1 Medical School, Faculty of Medicine, University of Zawia, Zawia, LBY; 2 Department of Family and Community Medicine, Faculty of Medicine, University of Zawia, Zawia, LBY; 3 Department of Pathology, Faculty of Medicine, University of Zawia, Zawia, LBY

**Keywords:** anxiety, depression, medical students, mental health, psychological distress, social media addiction

## Abstract

Introduction: Social media addiction (SMA) is considered a major public health issue due to the increasing negative effects on academic performance, social behavior, and psychological well-being. The risk of addiction is heightened by students' use of social media platforms for communication, relationships, entertainment, or academic purposes. The current work assessed the examined social media addiction as a determinant of psychological distress and academic performance among Libyan future medical doctors.

Methods: A cross-sectional study was conducted, and a self-reported questionnaire was utilized using Bergen Social Media Addiction Scale (BSMAS), alongside validated measures for depression using the patient health questionnaire (PHQ-9) scale, anxiety using the generalized anxiety disorder 7-item (GAD-7) scale, and internet usage patterns. The questionnaire was pretested (n = 60) and demonstrated good internal reliability (Cronbach’s α = 0.78-0.85). Medical students from their third academic year to the intern level were eligible to participate in the survey.

Results*:* Among 318 participants, the prevalence of SMA was 12 (4%), while 95 (30%) were identified at high-risk users. Chi-square analysis showed that SMA was significantly associated with the average daily time spent online, and symptoms of depression and anxiety, while no significant association was observed with sociodemographic variables. Multiple linear regression showed that daily internet use, time spent online, and mental health symptoms were strong predictors of BSMAS score.

Conclusion: This study found that SMA, depression, and anxiety are common among Libyan medical students. These findings highlight the relevance of incorporating digital wellness and mental health education into medical curricula, while longitudinal studies are needed to clarify causal pathways.

## Introduction

In recent years, interactive social communication systems, represented by major global platforms, have exploded in popularity, becoming a primary mode of communication, education, and social interaction [1,2]. The COVID-19 pandemic accelerated the rise of technology and internet services, especially social media, creating a new digital reality for society as a whole and students in particular [3]. According to global estimates, by 2028, the number of people using social media is expected to rise to over six billion compared to 2024, with over five billion, as social networking becomes the most popular activity worldwide [4].

In Libya, as of January 2025, roughly 86.3% of the population uses the internet, and approximately 6.40 million of them are active on social media, representing an 8.5% rise over the previous year. In addition, Facebook (Meta Platforms, Inc., Menlo Park, California, United States) is the dominant platform, used by 97.5% of internet users, followed by TikTok (ByteDance Ltd., Haidian, Beijing, China) [5]. However, usage rates often exceed population benchmarks due to multiple accounts' behavior and platform overlap. For students, social media and internet accessibility provide chances for information sharing, networking, and engagement. However, excessive, prolonged, and uncontrolled usage can result in social media addiction (SMA), a problematic behavioral pattern that impairs social, emotional, and academic functioning [6].

The criteria for SMA, which were taken from Griffiths’ (2005) model of behavioral addiction, include compulsive involvement, mood change, withdrawal symptoms, and interference with personal and academic pursuits [7]. SMA is associated with impulsivity and low self-esteem, as well as mental and psychological conditions like depression, anxiety, and obsessive-compulsive disorder [6,8]. It is also associated with reduced time spent on academic work and poorer academic performance [9,10]. In university settings, which involve a large scale of social media integration for communication, entertainment, and academic purposes, students may be at greater risk to develop SMA [10,11]. A recent study from Saudi Arabia reported that medical as well as non-medical university students are at high levels of problematic social media use, with significant impact on sleep, daily function, anxiety, and academic performance [11].

According to a recent meta-analysis encompassing 32 nations across seven distinct world regions, the pooled prevalence of SMA was 24%, with substantial variations by age, region, and diagnostic criteria [12]. Given the widespread use of social media in Libya, research into SMA and its determinants is increasingly critical, yet remains limited. Therefore, this study aimed to estimate the prevalence of SMA among medical students at the University of Zawia, Libya, and to examine its association with psychological distress and academic performance.

The findings of this study are expected to inform initiatives aimed at reducing excessive social media use and strengthening awareness within academic settings.

## Materials and methods

Study design and protocol

An institution-based cross-sectional study was conducted among medical students at the Faculty of Medicine, University of Zawia, Zawia, Libya, to assess the prevalence of SMA and its determinants. The study was approved by the Biosafety and Bioethics Committee of the Libyan Medical Research Center (approval number: NBC:018.H.24.36).

Study population, sampling, and sample size

Medical students from the third academic year through the intern level were eligible to participate. First- and second-year medical students were excluded because they were in introductory years. A convenience sampling method was used.

The sample size in the present investigation was obtained using the following equation: $n = \frac{Z^2 \, p \, (1 - p)}{d^2}$, where n is the required sample size, Z is the standard normal deviate corresponding to the desired confidence level (1.96 for 95% confidence), p is the estimated population prevalence, and d is the margin of error, taking into account of 55% population prevalence with a 95% degree of confidence, and 5% margin of error. The minimum required sample size was 267 participants. Given the use of a convenience non-probability sampling technique (due to logistical constraints and the absence of an institutional email system), the final sample obtained was 318, exceeding the required minimum.

Data collection

Data were collected between November 2024 and January 2025. An online questionnaire created with Google Forms (Google LLC, Mountain View, California, United States) was used for data collection. The survey link was circulated through commonly used student communication channels at the Faculty of Medicine (official Google Classrooms (Google LLC) and Telegram (Telegram Group Inc., Tortola, British Virgin Islands) class groups). The survey link included an electronic consent form that outlined the study’s goal, voluntary participation, and guaranteed confidentiality and data privacy.

Study tools and measures

The study questionnaire contained four main sections. The first section included participants' sociodemographic variables, such as age, gender, study year, levels of physical activity, current smoking status, and academic performance. The performance was measured by the grade of the recent academic year as excellent (85-100%), very good (75-84%), good (65-74%), or acceptable (60-64%). The second section assessed internet and social media use by asking about everyday use of the internet (average time and frequency) and the primary social media platform accessed (see Appendix A).

The following section used the six-item Bergen Social Media Addiction Scale (BSMAS) to assess SMA, the study's outcome variable [13]. This scale was designed as a one-dimensional scale with six items/indicators, which take into account salience, tolerance, mood modification, relapse, withdrawal, and conflict. Each question was scored with a five-point Likert scale ranging from very rarely (1) to very often (5), with the summative total determining the BSMAS raw score, with the BSMAS total score ranging from 6 to 30. SMA was indicated by a cut-off score of BSMAS ≥ 24. This threshold was chosen depending on the sensitivity-specificity analysis presented by Luo et al., which showed the best separation between problematic and non-problematic social media users [14]. Although this cut-off has not yet been approved specifically in the Arab or North African student populations, international research employing the BSMAS has adapted it very widely [15]. Applying this set threshold offers a standardized and comparable criterion for categorizing SMA risk in the absence of regional validation studies.

The last section of the questionnaire was used to assess psychological distress (i.e., anxiety and depression) as determinants of SMA. The anxiety symptoms were assessed using the generalized anxiety disorder seven-item (GAD-7) scale [16]. The GAD-7 consists of seven items, each of which was rated on a four-point Likert scale ranging from never (0) to nearly every day (3). The GAD-7 total score ranged from 0 to 21, with 0-4 indicating no/minimal anxiety, 5-9 indicating mild anxiety, 10-14 indicating moderate anxiety, and 15-21 indicating severe anxiety. The degree of depression symptoms was assessed using the Patient Health Questionnaire (PHQ-9) scale [17]. The PHQ-9 scale is a validated nine-item questionnaire with responses ranging from never (0) to nearly every day (3). The total score ranges from 0 to 27, with 0 indicating no depression, 1-4 indicating minimal depression, 5-9 considered mild, 10-14 considered moderate, 15-19 considered moderately severe, and 20-27 considered severe depression. 

The questionnaire was distributed on various official websites and social media platforms. The initial data from a pilot study with the first 60 participants were collected using IBM SPSS Statistics for Windows, version 26 (Released 2018; IBM Corp., Armonk, New York, United States). The internal consistency for the scales in the current study was high. Adjusted Cronbach’s alpha for the BSMAS was 0.81, alpha for the GAD-7 was 0.85, and alpha for the PHQ-9 was 0.78. 

Statistical analysis

The data was transferred to a Microsoft Excel sheet (Microsoft Corporation, Redmond, Washington, United States) before being exported to the R statistical computing platform version 4.2.1 (2023; R Foundation for Statistical Computing, Vienna, Austria, https://www.R-project.org/). Descriptive statistics, including frequencies and percentages for categorical variables and means with standard deviations (SDs) for numeric variables, were performed. Chi-square test was performed to compare the variables under investigation. Regression assumptions were evaluated prior to analysis. Multicollinearity was evaluated using the variance inflation factor (VIF) values (>2.5). Normality of residuals was checked using the Shapiro-Wilk test (P < 0.001) and Q-Q plots, which showed an acceptable linear pattern. Homoscedasticity was examined through residual-versus-fitted plots and Breusch-Pagan test (BP =15.54, P =0.49), indicating constant variance. A multiple linear regression model was used to examine the association between SMA (BSMAS score) and the following predictor factors: age, sex, physical activity, academic performance, frequency of internet use per day, average time spent online per day, depression severity, and anxiety severity. The strength of association was presented using the regression coefficient (β) and standard error (SE). Statistical significance for analytic tests was set at a P* *< 0.05 throughout the analysis.

## Results

Participant characteristics

Of the 318 medical students, 252 (79%) were female, and 164 (52%) were in the age group of 24-26 years. The majority of the participants were fifth-year students (n=134; 42%). About 126 (40%) achieved a very good grade in the recent year. However, a notable proportion (n=184; 85%) stated no physical activity, and 308 (97%) were non-smokers. The detailed students’ sociodemographic characteristics are summarized in Table 1.

**Table 1:** Sociodemographic characteristics of the participants (N = 318)

Variable	Frequency (Percentage)
Gender	
Female	252 (79%)
Male	66 (21%)
Age Group (years)	
21-23	133 (42%)
24-26	164 (52%)
27-29	21 (6%)
Medical School Level	
3^rd^ year	54 (17%)
4^th^ year	54 (17%)
5^th^ year	134 (42%)
Intern	76 (24%)
Grade of the recent year	
Excellent	91 (29%)
Very Good	126 (40%)
Good	90 (28%)
Accepted	11 (3%)
Physical Activity Levels	
No activity	184 (58%)
< 3 times\week	97 (30%)
3-5 times\week	28 (9%)
> 5 times\week	9 (3%)
Current Smoking Status	
No	308 (97%)
Yes	10 (1%)

Social media addiction

Based on the BSMAS scoring cut-off of >24, the estimated prevalence of SMA among the participating medical students was 12 (4%). About 95 (30%) study participants were at high risk of SMA with a score of 19 or above. In the SMA group, all students were female, with an average age of 24-26 years (58.3%), and in their fifth medical year at the time of data collection. However, there were no statistically significant differences between the SMA group and the non-SMA group in terms of sociodemographic characteristics (Table 2).

**Table 2:** Comparison of key characteristics and Internet use between the SMA Group and non-SMA Group based on the BSMAS with cut-off point of 24 * significant Chi-Square test with *P* < 0.05. SMA: social media addiction; BSMAS: Bergen Social Media Addiction Scale

Variable	SMA group (n=12)	non-SMA group (n=306)	
Gender			
Female	12 (100%)	240 (78.4%)	
Male	0 (0%)	66 (21.6%)	
Age Group (years)			
21-23	4 (33.3%)	129 (42.2%)	
24-26	7 (58.3%)	157 (51.3%)	
27-29	1 (8.4%)	20 (6.5%)	
Medical School Level			
3^rd^ year	2 (16.7%)	52 (28.8%)	
4^th^ year	1 (8.3%)	53 (39.5%)	
5^th^ year	7 (58.3)	127 (28.4%)	
Intern	2 (16.7%)	74 (3.3%)	
Grade of the recent year			
Excellent	3 (25%)	88 (28.8%)	
Very Good	5 (41.7%)	212 (39.5%)	
Good	3 (25%)	87 (28.4%	
Accepted	1 (8.3%)	10 (3.3%)	
Physical activity levels			
No activity	10 (83.4%)	174 (56.9%)	
< 3 times\week	1 (8.3%)	96 (31.4%)	
> 3 times\week	1 (8.3%)	36 (11.7%)	
Frequency of internet use/day			
< 2 times/day	0 (0%)		39 (12.7%)
> than 2 times/day	12 (100%)		267 (87.3%)
Average time spent/day *			
< 5 hours	2 (16.7%)		193 (63.1%)
> 5 hours	10 (83%)		113 (36.9%)

In terms of online timing, frequency, and social media platforms, approximately 279 (88%) students reported using the internet more than twice daily. This pattern was found in all groups, with no statistically significant differences (see Appendix B). Furthermore, 166 (52%) students spend between one and five hours every day online. However, the average time spent on the internet for the SMA group was more than five hours per day, with a significant statistical difference in comparison with the non-SMA group (P <0.05) (Table 2). 

**Figure 1:**
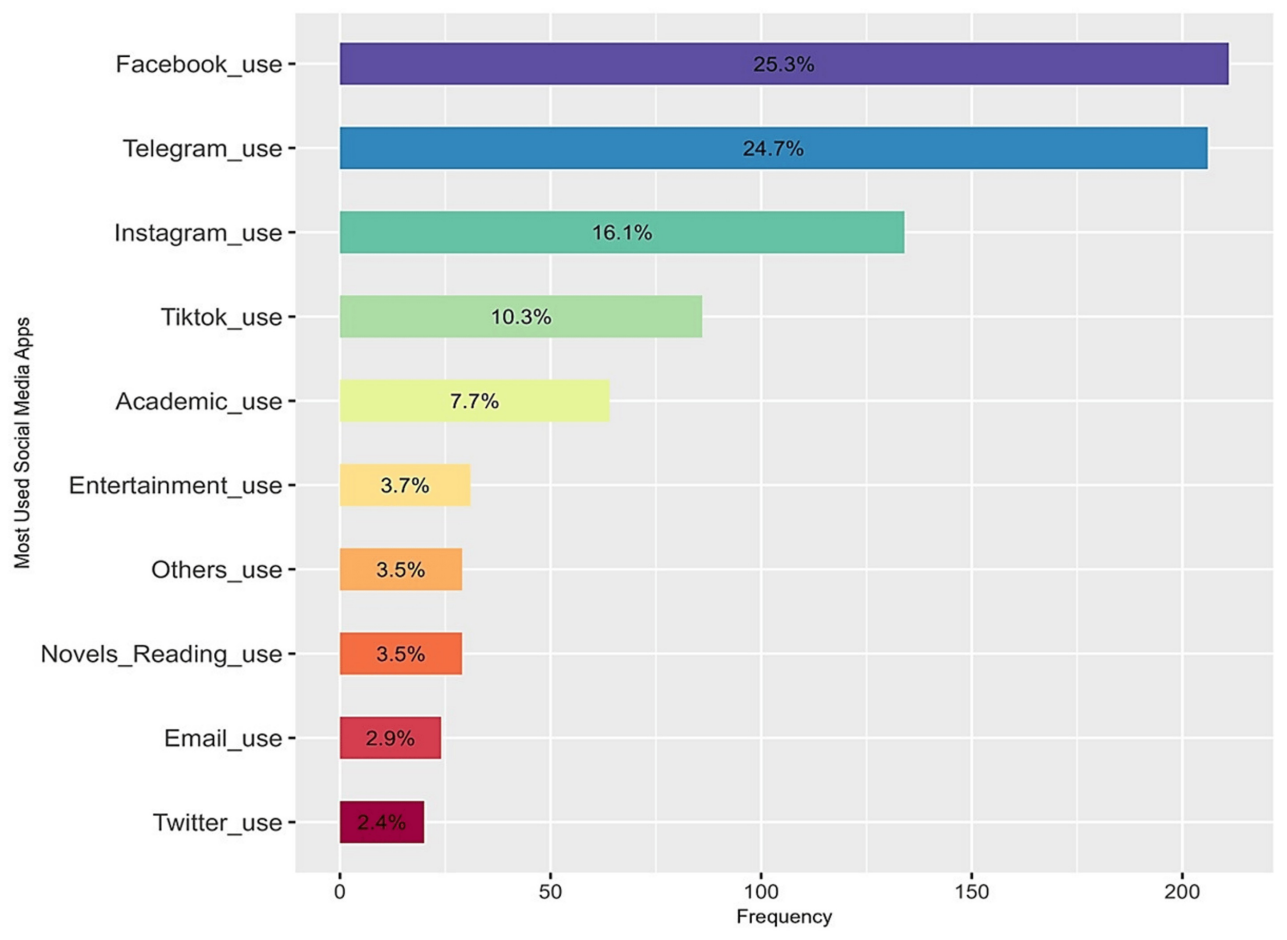
Prevalence of the frequently used social media platforms among the participants (N=318) Facebook was the most popular platform (n=211, 25.3%), followed by Telegram (n=206, 24.7%) and Instagram (n=134, 16.1%). Other platforms saw limited use.

The majority of participants identified Facebook, Telegram, and Instagram (Meta Platforms, Inc.) as their most utilized social media platforms, respectively (Figure 1). With regard to the SMA group, the most popular social media platforms are also Facebook (n=8, 20%) and Telegram (n=8, 20%), followed by TikTok (n=7, 17%) and Instagram (n=7, 17%).

Behavioral and mental health-related characteristics

**Figure 2:**
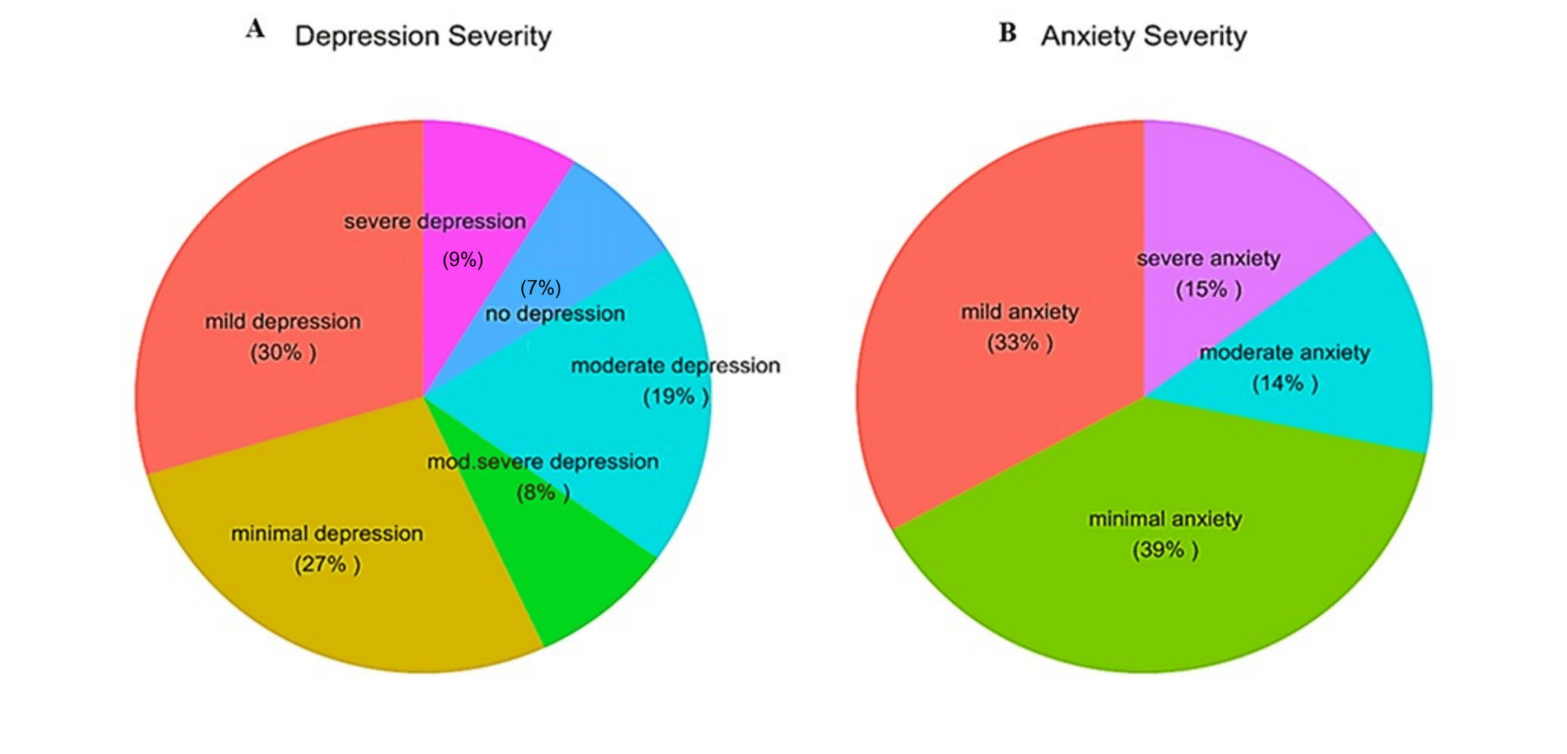
Distribution of participants (N=318) according to severity of anxiety and depression (A) Depression severity based on PHQ-9 scale of Depression; (B) Anxiety severity based on GAD-7 scale of Anxiety. PHQ-9: nine-item Patient Health Questionnaire; GAD-7: seven-item Generalized Anxiety Disorder

According to the PHQ-9 scale, about 114 (35.9%) participants had symptoms of depression that ranged from moderate to severe, with an average depression score of 8.4. Furthermore, about 195 (61.3%) students experienced anxiety symptoms ranging from mild to severe according to the GAD-7 scale (Figure 2). Compared with the non-SMA group, nine (75%) students in the SMA group (n = 12) exhibited severe depression and severe anxiety symptoms, with a statistically significant difference (P < 0.001) (Table 3).

**Table 3:** Distribution of psychological distress (anxiety and depression) based on the SMA status. SMA: social media addiction

Categories	Non-SMA Group (n =306), n (%)	SMA Group (n =12), n (%)	P value
Depression Categories			
No depression	23 (7.5%)	0 (0%)	<0.001
Minimal depression	87 (28.4%)	0 (0%)	
Mild depression	93 (30.4%)	1 (8.3%)	
Moderate depression	59 (19.3%)	1 (8.3%)	
Moderate to severe depression	25 (8.2%)	1 (8.3%)	
Severe depression	19 (6.2%)	9 (75.0%)	
Anxiety Categories			
Minimal anxiety	122 (39.9%)	1 (8.3%)	<0.001
Mild anxiety	105 (34.3%)	0 (0%)	
Moderate anxiety	41 (13.4%)	2 (16.7%)	
Severe anxiety	38 (12.4%)	9 (75.0%)	

Association analysis between BSMAS and other variables

In this study, the multiple linear regression analysis showed about 50.3% (R2 = 0.503, adjusted R2 = 0.46) of the variation within the BSMAS score is explained by the significant predictors in the model with a statistically significant overall model (P < 0.0001) (Table 4). There were, however, no significant associations identified between the BSMAS score and the sociodemographic variables (i.e., gender, age, most measures of physical activity, and medical study level) (P* *> 0.05).

**Table 4:** Multiple linear regression analysis showing the association predictors with SMA (BSMAS Score) among study participants (N= 318). β = standardized regression coefficient; *P < 0.05, **P < 0.01, ***P < 0.001. The R2 for the adjusted linear regression model was 0.46***. CI: confidence interval; SE: standard error; SMA: social media interval; BSMAS: Bergen Social Media Addiction Scale

Variable (s)	β	95% CI	SE	*P* value
Gender				
Male	Reference			
Female	-0.02	(-1.04, 0.99)	0.52	0.96
Age Group (years)				
21-23	Reference			
24-26	-0.62	(-1.74, 0.49)	0.56	0.27
27-29	0.10	(-1.89, 2.10)	1.01	0.92
Medical School Level				
3^rd^ year	Reference			
4^th^ year	-0.28	(-1.62, 1.05)	0.68	0.67
5^th^ year	-0.29	(-1.66, 1.07)	0.69	0.67
Intern	-0.25	(-1.94, 1.44)	0.86	0.77
Grade of the recent year				
Excellent	Reference			
Very Good	1.48	(0.49, 2.46)	0.50	0.003**
Good	1.34	(0.24, 2.44)	0.55	0.01*
Accepted	2.46	(0.19, 4.74)	1.15	0.03*
Physical activity levels				
No activity	Reference			
< 3 times/week	-0.08	(-0.95, 0.79)	0.44	0.8
3-5 times/week	0.30	(-1.14, 1.75)	0.73	0.6
> 5 times/week	0.65	(-1.70, 3.01)	1.19	0.5
Frequency of internet use/day				
1 time/day	Reference			
2 times/day	1.49	(-1.28, 4.27)	1.41	0.2
> 2 times/day	3.65	(1.02, 6.28)	1.33	0.006**
Average time spent/day				
30 minutes-1 hour	Reference			
1-5 hours	2.92	(1.28, 4.56)	0.83	0.0005***
> 5 hours	4.98	(3.27, 6.68)	0.86	<0.0005***
Depression Severity				
No depression	Reference			
Minimal	-0.03	(-1.72, 1.65)	0.85	0.9
Mild	1.20	(-0.61, 3.03)	0.92	0.1
Moderate	2.43	(0.44, 4.43)	1.01	0.01*
Moderate to Severe	3.15	(0.82, 5.48)	1.18	0.008**
Severe	4.17	(1.67, 6.67)	1.26	0.001**
Anxiety Severity				
Minimal	Reference			
Mild	1.55	(0.47, 2.64)	0.55	0.005*
Moderate	0.93	(-0.55, 2.42)	0.75	0.2
Severe	2.01	(0.29, 3.73)	0.87	0.02*

On the other hand, several factors showed significant associations with the outcome of interest. There was a statistical inverse association between the academic performance and the BSMAS score. Lower academic performance was associated with a higher BSMAS score compared to the reference group with excellent GPA (Grade Point Average) (Very Good Grade, β =1.48, P =0.003 versus Accepted Grade, β =2.46,* P* =0.03) (Table 4). Notable associations were also found with daily internet use; the group that used the internet more than twice a day had a significantly higher score (β = 3.65, *p* = 0.006) than those who used it once a day, while the group that used the internet twice a day did not reach statistical significance.

Time spent on the internet per day, using students who spent 30 minutes to one hour per day online as a reference group, those spending one to five hours showed significantly higher association with BSMAS scores (β = 2.92, P = 0.0005), while students spending more than five hours per day demonstrated an even greater increase in addiction score (β = 4.98, P < 0.0005) (Table 4). 

In terms of psychological distress, students with higher levels of depression showed a statistically significant correlation with higher BSMAS scores. Increasing depression severity was associated with a greater estimated coefficient (moderate: β = 2.43, *p* = 0.01; moderate-to-severe: β = 3.15, P = 0.008; severe: β = 4.17, P = 0.001) (Table 4). A similar association was found between anxiety level and BSMAS score; mild anxiety (β = 1.56, P = 0.005) and severe anxiety (β = 2.01, P = 0.02) are positively correlated with higher BSMAS scores (Table 4).

Our study also evaluated social media's impact on academic performance by examining its influence on attendance, job performance, and productivity. As presented in Table 5, nearly 130 (40.9%) participants perceived that social media had no impact on their academic attendance. Few claimed frequent or occasional attendance impairment due to social media use. Regarding the impact of internet use on job performance and productivity, respondents were almost evenly divided between reporting no effect (22%), a rare effect (n=70, 22%), or an occasional effect (n=92, 28.9%).

**Table 5:** Impact of social media use on attendance, job performance, and productivity (N= 318)

Parameters		Frequency (Percentage)	Cumulative Percentage
How much does social media use affect your attendance?	Not at All	130 (40.9%)	40.9
	Rarely	79 (24.8%)	65.7
	Occasionally	59 (18.6%)	84.3
	Frequently	35 (11%)	95.3
	Often	10 (3.1%)	98.4
	Always	5(1.6%)	100.0
How often does your job performance or productivity suffer because of the Internet use?	Not at All	70 (22%)	22.0
	Rarely	81 (25.5%)	47.5
	Occasionally	92 (28.9%)	76.4
	Frequently	46 (14.5%)	90.9
	Often	19 (6%)	96.9
	Always	10 (3.1%)	100.0

## Discussion

The study examined the prevalence and influencing factors of SMA among medical students at the Faculty of Medicine, University of Zawia, Libya. To the best of our knowledge, this is among the first studies to investigate SMA among medical students in Libya. The BSMAS assessment revealed that SMA significantly affected 12 (4%) participants, and 95 (30%) participants exhibited significant risk indicators. Facebook, Telegram, and Instagram were the preferred social media platforms. The study found that SMA is significantly linked to the degree of depression and anxiety symptoms, excessive internet usage, and low levels of academic performance.

The BSMAS-based SMA prevalence in this study matches the 6% rate identified by a study with Turkish medical students [18], but it is lower than what researchers found in Egypt [19], Morocco [20], and Saudi Arabia [21]. The observed differences arise from cultural and academic aspects, as well as geographic elements and diagnostic tool variance, adjustments, and threshold settings. The impact of regional influence on SMA was also demonstrated by a large-scale meta-analysis that revealed that prevalence reached 31% in collectivist countries but remained noticeably lower at 8% in Western/Northern Europe, 15% in North America, 31% in Asia, 37% in Africa, and 29% in the Middle East [12].

In terms of sociodemographic features, we did not find any significant link between SMA and gender, age group, or academic year, which is similar to the findings of Sayili et al. [18] and Toumari et al. [22], while some studies have indicated gender disparities [13,21,23]. These contradicting findings imply that contextual elements like population, geography, and timing affect connections, calling for more research.

One of the keys finding of this study was the strong positive association between SMA and symptoms of depression and anxiety. Notably, about nine of the 12 (75%) participants in the SMA group displayed significant signs of both conditions. This observation aligns with the systemic examination conducted by Keles et al., which found an association between adolescent social media use and higher rates of depression, anxiety, and psychological distress [6]. Supporting this, Shensa et al. [24] and Alfeya et al. [21] also identified higher rates of depression among regular social media users. Nevertheless, future longitudinal studies are required to fully understand the complexity. and direction of these connections, as well as any potential mediating or moderating factors.

Another significant finding of this study was the inverse association between SMA and academic performance. Students with lower academic achievement (e.g., "Good" and "Accepted" grades) showed significantly higher BSMAS scores compared to those with "Excellent" grades. This implies that excessive usage of social media may have an adverse influence on academic achievement, whether through fragmenting attention, straining cognitive capacity, or reducing study time. This inverse relationship confirms other research showing the negative effects of smartphone addiction and social media on academic performance.

Additionally, some studies have reported a strong negative association between the usage time and severity of social media use and academic performance, such that higher levels of usage are associated with greater academic difficulties [22,25,26]. This insight actually seems to be universally applicable, given its validity in both East Asian and Middle Eastern cultures [21,27]. Even though our findings suggest that SMA increases the risk of academic underperformance, further research is still necessary to fully understand the mechanisms and potential mediators, such as reward system dysregulation, inefficient multitasking, or sleep disturbance.

Internet usage patterns were shown to have an additional important influence. Significantly higher BSMAS scores were shown by students who utilized the internet more than twice a day and for more than five hours. This observation is reinforced by previous studies, which demonstrated that excessive internet engagement is a strong predictor of SMA [18,22,25,26,28]. In our study, excessive internet use for social networking rather than academic purposes is likely to result in poor academic performance and intellectual impairment.

Our results also showed that Facebook is the most popular platform, followed by TikTok, Instagram, and Telegram, even though other previous regional studies have shown WhatsApp as the most prevalent platform [21,25,26]. Also, the platforms' educational and communicative values, along with entertainment purposes, were confirmed [20,29]. Even though our study did not examine the precise motivations for utilizing these platforms, it showed that access to social media and the internet did adversely impact participant productivity, attendance, or job performance. Nevertheless, future studies should establish the specific objectives for using social media within the cohort group.

In light of the observed association between SMA, psychological distress, and academic performance in the present study, the recent launch of Libya’s National Mental Health Strategy (2024-2030) by the Ministry of Health in collaboration with WHO provides an important policy framework for strengthening prevention, early detection, treatment, and psychosocial support for mental health conditions [30]. Although the strategy does not specifically address digital or behavioral addictions, its focus on early detection and community mental health aligns with the need to develop interventions that promote digital literacy, healthy internet use, and improved mental health awareness among university students.

This study offers insightful information and has several important strengths. To our knowledge, it is among the earliest investigations of SMA among medical students in Libya, addressing a major regional research gap. The use of validated instruments (BSMAS, GAD-7, PHQ-9), an adequate sample size, and multivariable regression analysis strengthens the reliability and interpretability of the study findings. Examining both psychological and academic correlates provides a more comprehensive understanding of SMA in this population.

Nonetheless, several limitations should be acknowledged. First, it is not possible to establish a causal relationship between SMA and its elements because of the cross-sectional design. Second, reliance on self-reported measures may have enhanced potential recall and social desirability biases, which could affect data accuracy. Third, the sample was drawn from a single institution, which may restrict the findings' generalizability to broader populations in Libya. Additionally, because the survey was distributed online through class Telegram groups and Google Classrooms, non-response bias cannot be completely ruled out. Although the participation was voluntary, students who were less engaged with online academic platforms may have been underrepresented. To overcome these constraints and improve the validity of the results, future studies should use multi-site sampling, objective measurements, and longitudinal approaches.

## Conclusions

Among university students in Libya, this is the first comprehensive SMA evidence. Although the total prevalence was relatively low, almost one-third of students were highly at risk of SMA. Depression and anxiety symptoms, excessive internet use, and poor academic performance were all substantially associated with SMA. These findings underscore the importance of addressing problematic social media use within the university population and highlight the need for integrating digital well-being and mental health education into medical training. Given the cross-sectional design, longitudinal studies are necessary to clarify pathways and to guide the design of successful preventive and intervention plans.

## Appendices

Appendix A: study questionnaire 

**Table 6:** Survey of social media addiction as a determinant of psychological distress and academic performance among the participants

PART 1: SOCIODEMOGRAPHIC DATA											
Student (ID)											
Age											
Gender			Male								
			Female								
Study Year			3rd year medical student				4th year medical student				
			5th year medical student				Internship period				
Satisfaction With Major Study			Good								
			Moderate								
			poor								
Grade Average			Excellent (> 85%)				Very good (>75-<85%)				
			Good (>65-<75%)				Accepted (>60-<65%)				
			Failed (<60%)								
Physical Activity Level			No activity				3 >times per week				
			3 to 5 times per week				>5 times per week				
PART 2: SOCIAL MEDIA ADDICTION SCORING											
How often?		Very rare (0)		Rare (1)		Sometimes (2)		Often (3)			Very often (4)
You spend a lot of time thinking about social media or planning how to use it											
You feel an urge to use social media more and more.											
You use social media in order to forget about personal problems.											
You have tried to cut down on the use of social media without success											
You become restless or troubled if you are prohibited from using social media.											
You use social media so much that it has had a negative impact on your job/studies											
PART 3: TYPE & TIME OF INTERNET USE FOR NON-STUDYING OR NON-WORKING PURPOSES											
Time For Internet Use	Total average time spent online use per day								30min-1hour		
									1-5hours		
									>5hours		
	Frequency of internet use per day								1 time\day		
									2times\day		
									More than 2time\day		
Purpose Of Internet Usage	How frequent of using? (Rarely (0), Neutrally (1), Frequently (2), Very Frequently (3))										
	Academic				Tweeter					Emails	
	Facebook				Instagram					Telegram	
	TikTok				Entertainment					Novels & readings	
	Others (specify)……………………………………………………………………										
PART 4: NEGATIVE IMPACT OF SOCIAL MEDIA ON MEDICAL STUDENTS (PSYCHOSOCIAL)											
Over the last two weeks, how often have you been bothered by the following problems? ( o Not at all, o Several days, o More than half day, o Nearly everyday											
Anxiety (GAD7-Scale)	Feeling nervous, anxious, or on edge										
	Not being able to stop or control worrying										
	Worrying too much about different things										
	Trouble relaxing										
	Being so restless that it is hard to sit still										
	Becoming easily annoyed or irritable										
	Feeling afraid, as if something awful might happen										
Depression (PHQ9-scale)	Little interest or pleasure in doing things										
	Feeling down, depressed, or hopeless										
	Trouble falling or staying asleep, or sleeping too much										
	Feeling tired or having little energy										
	Poor appetite or overeating										
	Feeling bad about yourself or that you are a failure or have let yourself or your family down										
	Trouble concentrating on things, such as reading the newspaper or watching television										
	Moving or speaking so slowly that other people could have noticed. Or the opposite being so fidgety or restless that you have been moving around a lot more than usual										
	Thoughts that you would be better off dead, or of hurting yourself										
ACADEMIC PERFORMANCE Does not apply (0), Rarely (1), Occasionally (2), Frequently (3), Often (4), Always (5)											
How often does your job performance or productivity suffer because of the Internet?											
How does the addiction affect your attendance											

Appendix B

**Table 7:** The most used online applications in the SMA group SMA: social media addiction

Social Media Platforms	Frequency (Percentage)
Facebook	8 (19.5%)
Telegram	8 (19.5%)
Instagram	7 (17.1%)
TikTok	7 (17.1%)
Academic use	4 (9.8%)
Other uses	3 (7.3%)
Novel reading	2 (4.9%)

**Table 8:** Frequency and average time spent on Internet among the participants *significant Chi-Square test with P <0.05 SMA: social media addiction

Variable	Overall (n=318), n (%)		SMA Group (n=12), n (%)	Non-SMA Group (n=306), n (%)	
Frequency of internet use/day					
1 time/day	9 (3%)		0 (0%)	9 (2.9%)	
2 times/day	30 (9%)		0 (0%)	30 (9.8%)	
> than 2 times/day	279 (88%)		12 (100%)	267 (87.3%)	
Average time spent/day *					
30 minutes to 1 hour	29 (9%)	0 (0%			29 (9.5%)
1-5 hours	166 (52%)	2 (16.7%)			164 (53.6%)
> 5 hours	123 (39%)	10 (83%)			113 (36.9%)
